# The construction of “official outlaws”. Social-psychological and educational implications of a deterrent asylum policy

**DOI:** 10.3389/fpsyg.2015.00382

**Published:** 2015-04-08

**Authors:** Margarita Sanchez-Mazas

**Affiliations:** Sciences of Education Group Intercultural Relations in Education, Faculty of Psychology and Sciences of Education, University of GenevaGeneva, Switzerland

**Keywords:** failed asylum seekers, social assistance, voluntary return, asylum policy, education, children's rights, learned helplessness, psychological reactance

With the increasing numbers of immigrants seeking to obtain political asylum, the receiving conditions and the deportability of refused asylum seekers have emerged as major issues. Concern with these questions has been addressed through renewed asylum policies involving expeditious processing of applications, tight restrictions upon the right to work, removal of support or detention for failed asylum seekers. These asylum policies are intended to reduce alleged “pull factors” through the use of the threat of destitution as a deterrent against asylum seeking (Da Lomba, 2006). They also aim at reducing the number of asylum seekers that remain in the territory despite having been rejected as refugees. In this regard, the deterioration of the conditions of reception is seen across the EU as one cornerstone of a “voluntary departure” policy (Fox O'Mahony and Sweeney, 2010). In this article, we examine the case of Switzerland, where the suppression of social assistance for rejected asylum seekers was intended to support such a policy. We report data from a field study showing how, in this country, this policy induces institutional practices that prevent, rather than promote, failed asylum seekers to leave the territory. We also discuss some implications of the Swiss system in the realm of education, suggesting that the institutional constraints imposed by the asylum policy jeopardize the implementation of the recognized unconditional right of children to education.

## The Swiss Federal asylum policy

The Swiss government decision (2004) to remove the social assistance for rejected asylum seekers was first enforced through its inclusion in a comprehensive budgetary saving program approved by popular vote (FF, 2003, pp. 5091–5286). This decision was opposed by most of the human rights defenders, voluntary associations and social movements with the view that its enforcement was conducive to destitution or increasing criminality. The authorities of the Swiss cantons were to follow and to support the costs of the federal requirements. The interplay between the federal and the cantonal prerogatives in Switzerland created disparities in the implementation of the legal provisions—namely the introduction of an “emergency aid”—that followed the social assistance suppression. The solutions thus varied locally according to the political climate and the diverse traditions among the Swiss cantons of welcoming foreigners and offering asylum to political or religious refugees (Vuillemier, 1992; Green et al., 2011). This contrasting reality is often obscured by the important coverage given to the Swiss political movements against immigrants who use the instruments of the direct democracy, such as popular initiatives, in their strategy to conquer power through national xenophobic campaigns (Papadopoulos, 1991; Gentile and Kriesi, 1998; Tabin, 1999).

The system of emergency aid is based on the unconditional right to a minimum coverage of basic living needs in order to prevent destitution (Article 12 of the Swiss Federal Constitution). Used mainly by individuals in emergency situations up to 2004, it has been set up for the whole group of rejected asylum seekers as a result of denying them social aid. The cantonal implementations include compulsory accommodation in collective centers isolated from the urban population but there is a great discrepancy over how the emergency shelters are organized: Some are quasi concentration regimes (systematic entrance checks, obligation of presence, prohibition of visits), other close during the day, even in winter. Despite some slight local improvements in favor of the residents, the cantonal structures are designed in order to strengthen the residents' dependency (vouchers for food and toiletries) and the control of the authorities in all aspects of everyday life. Moreover, people have to regularly renew the emergency assistance by presenting themselves at the migration offices (sometimes every 2 or 5 days), where most often they experience pressures to leave, intimidations, as well as derogative or even humiliating treatments. These conditions maintain asylum seekers in a state of controlled illegality and affect negatively the experience of both the targeted group and the agents of this policy.

## The institutional construction of invisibility

Our field research (Sanchez-Mazas, 2011), conducted in four Swiss cantons (2 German-speaking and 2 French speaking areas), reveals that the system of emergency aid in the framework of a deterrent asylum policy turns out to be an instrument of pressure and discouragement that shapes the experience of failed asylum seekers in such a way that most of them do not choose to return to their home states. Through a series of interviews among asylum specialists (*N* = 39) and asylum seekers concerned by the new regime (*N* = 32), we could elicit the perspectives and experience of these non-admissible, yet officially registered, migrants and those of public officials, social workers, and members of voluntary organizations. Most often, these agents reported that this mandatory policy not only was ineffective at promoting departures but also constrained institutional practices viewed in contradiction with their mission and their values. Contrary to the expected expansion of the number of departures, new and unforeseen obstacles to returns have been identified as stemming from this very policy. Indeed, our analyses show that most of the targeted individuals actually went out of administrative controls, while remaining on the territory in an irregular manner.

We identified several social psychological impediments that might explain this reverse and unpredicted effect. We focus here on two contrasting types of social psychological processes induced by institutional practices aimed at producing deterrence effects through pressures to leave and deterioration of living conditions. The first reaction frequently encountered among our respondents is concerned with the willingness to make a voluntary depart: It heightens a fundamental motivation to restore control against attempts to constrain the behavior and pressures to submit. This *psychological reactance* (Brehm, 1993) is expressed in terms of determination to stay in order to resist those institutional practices that impinge on individual freedom and autonomy. One of the migrants interviewed in our study (Sanchez-Mazas, 2011, p. 228) put it this way: “*The more people have their back to the door, the more they are driven to the ultimate sacrifice: remaining whatever happens. In a more liberal system, it would be more voluntary returns*.” Together with other comments collected, this example shows how the pressure to comply paradoxically becomes a factor that reinforces the individual's determination to stay at all costs. Hence, people do not stay *despite* the pressures to leave, but, rather, *because* of them.

Conversely, the second reaction results from lasting aversive conditions that make people incapable to develop a project. The deterrent policy pursued by the Swiss government heightens failed asylum seekers vulnerability by inducing weakening effects that can be conceptualized in terms of *learned helplessness* (Peterson et al., 1995). This is clearly expressed by one of our respondents: “*We despise ourselves because we are despised. This system leads people to self-destruction, this policy makes people to destroy themselves. In addition, it is counterproductive because this way we are not able not leave*.” Likewise, another asylum seeker argued that “*Well, the principle is that people leave but the reality is that people are so much deprived of their will, of their capacity, that they become amorphous. Many of them stand around doing nothing, like nailed down*” (Sanchez-Mazas, 2011, p. 229).

In analyzing these mechanisms, we propose the notion of “construction of invisibility” to refer to institutional practices triggering the disappearance of the targeted persons into clandestinity as a result of an induced increased willingness to stay or incapacity to leave. Such a process can be related to the wider phenomenon of the “legal production of illegality” that has been recently addressed on the area of contemporary migration (De Genova, 2002). The alleged efficiency of the state action in reducing the number of registered asylum seekers overstaying after being rejected stems primarily from the persons' suppression from the asylum statistics. In this way, the Swiss government could claim the success of a policy that produces illegality and destitution among those who have been described as “the most legally and socially disadvantaged people in western societies” (Castles and Davidson, 2000, p. 73). Moreover, it could pursue this policy (that first targeted mostly young men, whose application was rejected before thorough examination), by extending the decision to withdraw social assistance to all refused asylum seekers (2008), including families whose “institutional invisibilization” is problematic.

## A major challenge for education

The *Convention on the Rights of the Child* (ratified by Switzerland in 1997) makes primary education compulsory and available free to all. This poses the challenge for contemporary democracies of fully implementing this right when granted to children whose parents are staying in the country in an irregular manner (Laubentahl, 2011; Vandenhole et al., 2011). The extension of this right to children without legal status represents a reversal of the historical positions between parents and children in the migration in Switzerland. While in previous years temporary legal migrants where constrained either to hide their children or to leave them in the home country, the present relegation of migrants to a no-rights zone makes children the unique bearers of a right to insertion into society. But this right remains fragile for a population school inherently unstable and who cannot, for political reasons, fully integrate the receiving society. In the emergency assistance system especially, the overcrowding and insecure conditions of the centers are highly inappropriate for school working and involvement. Furthermore, the lack of autonomy, as well as forms of adults' “infantilization” through the institutional handling of the residents, poses a threat to the parental image and the educational relationships (Moro and Barou, 2003).

In the Swiss academic world, the lack of institutional support for dealing with the educational needs of these children is a direct outcome of an asylum policy which denies any effort that could contradict the view that rejected asylum seekers must be discouraged to stay. This leaves the staff unprepared to receive children from very diverse cultural and linguistic contexts and who are often in conditions of extreme vulnerability upon arrival at school. Teachers worry about previous “deschooling,” problems of illiteracy or severe educational delays they are ill equipped to manage (Sanchez-Mazas, 2013). The feeling of being abandoned by the institution induces a sense of helplessness or guilt. Negative stereotyping and pupils' marginalization within the classroom are frequent outcomes of this situation in a political context that targets asylum seekers as undeserving and unwanted foreigners (Schmidlin et al., 2006).

## Conclusion

Under the euphemistic label (Bandura, 1999) of “individual and institutional incitation,” the primary purpose of the Swiss authorities was to “increase voluntary disappearances as a result of discouragement” (Gerber and Führer, 2000, p. 7; Tafelmacher, 2009). Besides the construction of an invisible population within a democratic State, the Swiss asylum policy leads to the creation of a category of people who depend on and are under the control of the very authority that tries to deport them. In this way, it turns into “official outlaws” (Achermann, 2009) those who remain in the country in conditions of total deprivation of rights and under the threat of being arrested or subject to forced departure for illegal stay. For some people, this leads to a process of disappearance, whereby they end up in a social vacuum by being excluded from institutions and from any legal existence whatsoever. For others, namely families with children, this introduces a tension between their illegal status and their children right to attend school that threatens the human right to receive education.

Unlike the interpersonal form of invisibility that is intended to actively show disrespect (Honneth, 2001), the institutional invisibility following the asylum deterrent policy represents a de-institutionalization of existence leading to social death (Renault, 2004). The dilemmas and moral conflicts expressed by most of the agents of this policy suggest that the dehumanization process these groups are undergoing would not simply be interpreted as resulting from individual stereotyping, prejudice and discrimination (for a review, see Haslam and Lougham, 2014). Our research helps grasping some structural factors that play a role in a dehumanization process beyond, or even *against*, individual attitudes. By uncovering the institutional production of invisibility through constrained social practices and organizational arrangements, it may contribute to capture the consequences of the sociopolitical processes and the institutional factors that, in Western contemporary democracies, construe the group of stateless people, which Arendt (1951/2004) marked out as having lost all other qualities and relationships, except that they are still human.

### Conflict of interest statement

The authors declare that the research was conducted in the absence of any commercial or financial relationships that could be construed as a potential conflict of interest.
